# [^18^F]F-PSMA-1007 Radiolabelling without an On-Site Cyclotron: A Quality Issue

**DOI:** 10.3390/ph14070599

**Published:** 2021-06-22

**Authors:** Valentina Di Iorio, Stefano Boschi, Anna Sarnelli, Cristina Cuni, David Bianchini, Manuela Monti, Giancarlo Gorgoni, Giovanni Paganelli, Federica Matteucci, Carla Masini

**Affiliations:** 1 IRCCS Istituto Romagnolo per lo Studio dei Tumori “Dino Amadori” IRST, 47014 Meldola, Italy; anna.sarnelli@irst.emr.it (A.S.); cristina.cuni@irst.emr.it (C.C.); david.bianchini@irst.emr.it (D.B.); manuela.monti@irst.emr.it (M.M.); giovanni.paganelli@irst.emr.it (G.P.); federica.matteucci@irst.emr.it (F.M.); carla.masini@irst.emr.it (C.M.); 2 Department for Life Quality Studies, University of Bologna, 47921Rimini, Italy; stefano.boschi@unibo.it; 3 Radiopharmacy & Cyclotron Department, IRCCS Ospedale Sacro Cuore-Don Calabria, 37024 Negrar di Valpolicella, Italy; giancarlo.gorgoni@sacrocuore.it

**Keywords:** prostate cancer, PSMA, [^18^F]F-PSMA-1007, [^18^F]F^-^, quality assurance, PET Radiopharmacy

## Abstract

Radiopharmaceuticals targeting the prostate-specific membrane antigen (PSMA) has become the gold standard for PET imaging of prostate cancer. [^68^Ga]Ga-PSMA-11 has been the forerunner but a [^18^F]F-PSMA ligand has been developed because of the intrinsic advantages of Fluorine-18. Fluorine-18 labelled compounds are usually prepared in centers with an on-site cyclotron. Since our center has not an on-site cyclotron, we decided to verify the feasibility of producing the experimental ^18^F^-^labelled radiopharmaceutical [^18^F]F-PSMA-1007 with [^18^F]F^-^ from different external suppliers. A quality agreement has been signed with two different suppliers, and a well-established and correctly implemented quality assurance protocol has been followed. The [^18^F]F^-^ was produced with cyclotrons, on Nb target, but with different beam energy and current. Extensive validation of the [^18^F]F-PSMA-1007 synthesis process has been performed. The aim of this paper was the description of all the quality documentation which allowed the submission and approval of the Investigational Medicinal Product Dossier (IMPD) to the Competent Authority, addressing the quality problems due to different external suppliers. The result indicates that no significant differences have been found between the [^18^F]F^-^ from the two suppliers in terms of radionuclidic and radiochemical purity and [^18^F]F^-^ impacted neither the radiochemical yield of the labelling reaction nor the quality control parameters of the IMP [^18^F]F-PSMA-1007. These results prove how a correct quality assurance system can overcome some Regulatory Authorities issue that may represent an obstacle to the clinical use of F-18-labelled radiopharmaceuticals without an on-site cyclotron.

## 1. Introduction

In the last years, the prostate-specific membrane antigen (PSMA) has become the gold standard for PET imaging of prostate cancer [1,2]. One of the first radiopharmaceuticals for this purpose was [^68^Ga]Ga-PSMA-11, which has been included in national and european prostate cancer guidelines [3] based on the diagnostic superiority of [^68^Ga]Ga-PSMA-11 compared to [^11^C]C-choline and [^18^F]F-choline already reported in the scientific literature [4].

[^68^Ga]Ga^3+^ has several advantages: from a chemical point of view, as a trivalent metal cation, it can be easily linked to target-specific molecules through the use of bifunctional chelators. Moreover, availability of [^68^Ge]Ge/[^68^Ga]Ga generators also enables small production sites without cyclotron to prepare PET tracers [5].

On the other hand, [^68^Ga]Ga^3+^ has also disadvantages such as limited production due to the capacity of the generators (not more than 1850 MBq), thus limiting the number of patients who can be imaged and high positron energy (about 1899 KeV) that can affect the quality of imaging PET. Furthermore, the rather short half-life of Gallium-68 (67.63 min.) does not allow for late acquisition of images [6].

For these reasons, the clinical research aimed at ^18^F-labeled compounds, first of all [^18^F]F-PSMA-1007 [7], that, compared to Gallium-68, allow simpler management of radiopharmaceuticals and higher quality of imaging due to lower positron endpoint energy (about 633 KeV), a longer half life (about 110 min), the possibility of large scale production and distribution, and a very low accumulation in the urinary system, that makes [^18^F]F-PSMA-1007 suitable to identify small lesions in the pelvis or for local recurrence. [^18^F]F-PSMA-1007 is usually prepared extemporaneously in a radiopharmacy equipped with cyclotron [8].

In 2020 Italian Regulatory Agency AIFA approved our clinical trial “Experimental study to evaluate the impact of 18F-PSMA PET/CT in the management of patients with prostate cancer” EudraCT 2019-002000-41. The primary objective of this diagnostic trial was to evaluate the sensitivity of [^18^F]F-PSMA-1007 PET/CT defined as the ratio between the number of [^18^F]F-PSMA-1007 PET/CT positive patients and the number of prostate cancer patients with biochemical relapse and negative standard imaging. The clinical trial includes the administration of a single intravenous injection of the radiopharmaceutical [^18^F]F-PSMA-1007 (150–250 MBq) prepared in our Radiopharmacy. In order to use a radio-pharmaceutical in human applications, this radiopharmaceutical has to be produced and its quality ascertained under Good Manufacturing Practice (GMP) or National Regulations. In Italy, the reference quality assurance system is “Norme di Buona Preparazione dei Radiofarmaci per Medicina Nucleare (NBP-MN)” [9], which can be assumed as a GMP-like quality assurance system.

To submit the clinical study to the Competent Authority an Investigational Medicinal Product Dossier (IMPD) according to EMA guideline has been drafted [10]. This guideline aims to address the documentation on the chemical and pharmaceutical quality of investigational medicinal products IMPs, included radiopharmaceuticals, to ensure the quality, safety and efficacy of these IMPs. Since our center has not an on-site cyclotron, the principal hurdle to overcome was to describe the organization, the procedure and the quality controls to guarantee quality issues of the final product even in presence of an external supply of [^18^F]F^-^. The aim of this paper is the description of all the quality documentation which allowed the submission and approval of the IMPD to the Competent Authority, addressing the quality problems due to different external suppliers.

## 2. Results

IMPD should be provided in a clearly structured format according to the Guideline [10] and should include the most up-to-date information relevant to the clinical trial available at the time of submission of the clinical trial application. It essentially consists of two parts, the first dedicated to the drug substance and one dedicated to the investigational medicinal product under test.

### 2.1. Drug Substance (2.2.1.S)

In the Section 2.2.1, 2.2.1.S of the IMPD, detailed information about drug substances (DS) is required; for this study, two drug substances have been identified: the ligand PSMA-1007 and the precursor [^18^F]F^-^.

#### 2.1.1. PSMA-1007

All the information required for PSMA-1007 were detailed in the quality document “Chemistry Manufacturing and Controls” (CMC) drafted by the supplier. The CMC document is continuously updated by the producer with the last information for example about the results of the stability studies for each time point check. Moreover, the results of the quality controls of each batch of PSMA-1007 are reported in the Certificate of Analysis (CoA) that is supplied with each batch. It is the user’s responsibility to check these documents, verify the correct storage conditions and verify the expiration date before the use of the ligand for radiolabelling.

#### 2.1.2. [^18^F]F^-^

Since our radiopharmacy doesn’t have an on-site cyclotron, for the supply of [^18^F]F^-^ we have been looking for a supplier compliant with the European Pharmacopoeia Monograph 01/2011:2390 “FLUORIDE (18F) SOLUTION FOR RADIOLABELLING” [11]. Based on these conditions, we signed a quality agreement with Supplier 1 for experimental setup and later with Supplier 2 for further clinical use (see Section 4.1.2, Reagents), as reported in IMPD.

The delivery was performed by an authorized transporter; the vial of [^18^F]F^-^ was collected at release and delivered directly to IRST Radiopharmacy in an average time of 2 h. The activity range provided by the suppliers is 12.6–30.5 GBq.

The quality of fluoride has been a major concern especially about impurities (Section 2.2.1, S.3.2 of the IMPD) which mainly depend on the production process and could potentially represent an undue dose for the patient.

The concomitant nuclear reactions are mainly due to irradiation with protons of the small percentage of H_2_^16^O contained in H_2_^18^O, ^16^O (p, α) ^13^N, which produces unreactive ^13^N (T_1/2_ = 9.96 min) with a very short half-life, as well as the nuclear reactions that occur on the Havar foils, since the Nb body should be considered as inert.

The impurities coming from the irradiation of Havar foils can be predicted based on the analysis of the possible cross-sections of the reactions with protons on the materials exposed to the beam, in a defined range of beam energy (Table 1 [12]).

The contribution of these impurities was experimentally measured in the various phases of the synthesis and appears to be of the order of KBq in the investigational medicinal product under test, therefore two orders of magnitude (10^−6^) lower than the activity of the radionuclide of interest.

The radionuclidic purity is verified by the producer at EOB and the results of quality controls are reported in the certificate of analysis which is supplied for each batch. We also emphasize that transport cannot affect the nature of the [^18^F]F^-^ either chemically or physically. Before using for radiolabelling, we check the certificate of analysis in each part. Moreover, during the validation of the suppliers, we carried out all the quality controls reported in the Monograph 01/2011:2390 [11] before start of synthesis, to compare our results with those of the supplier. All the results have been compliant with the CoA of the supplier (Table 2).

### 2.2. Investigational Medicinal Product under Test (IMP)

#### 2.2.1. Description and Composition of the IMP

IMP consists of a water/ethanol solution of [^18^F]F-PSMA-1007 with an activity range of 7000–18,200 MBq at End of Synthesis (EOS) which in this case is also considered Activity Reference Time (ART). The final volume is about 23 mL and the radioactive concentration is between 300 and 791 MBq/mL. [^18^F]F-PSMA-1007 is formulated as a multidose drug with the components described in Table 3.

#### 2.2.2. Description of the Manufacturing Process and Process Controls

**Figure 1:**
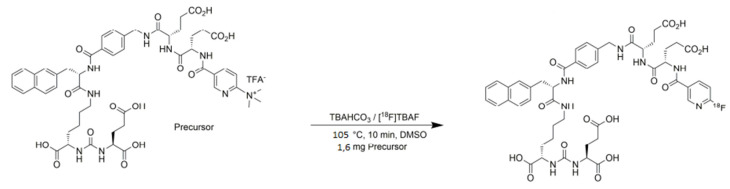
Radiosynthesis route of [^18^F]F-PSMA-1007.

For the radiolabelling of [^18^F]F-PSMA-1007 an automated synthesis module AllInOne (TRASIS-SA, Ans, Belgium) has been used. The PSMA-1007 substrate is fluorinated by the nucleophile [^18^F]F^-^ with a single step S_N_2 mechanism. The scheme of the radiosynthesis of [^18^F]F-PSMA-1007 is shown in Figure 1.

**Figure 2:**
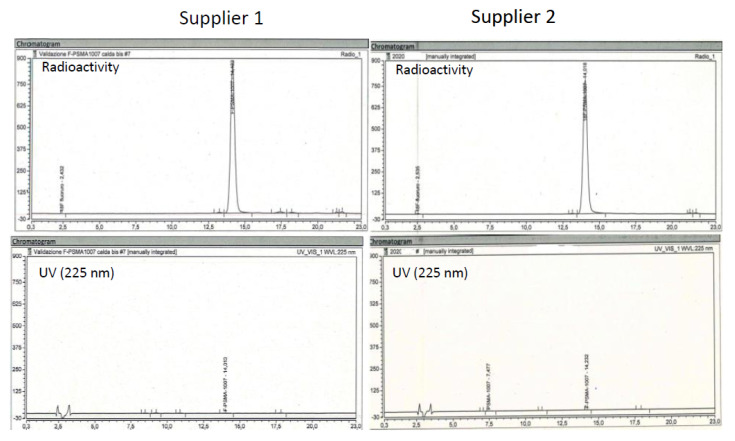
Relevant chromatogram of IMP [^18^F]F-PSMA-1007 obtained with [^18^F]F^-^ from the two suppliers.

Typical radio and UV chromatograms of synthesis performed with [^18^F]F^-^ from each supplier is shown in Figure 2. Typical synthesis time is 40 min. The overall mean synthesis yield was 48.6 ± 8.1% non-decay corrected (*n* = 44). The mean synthesis yield obtained with [^18^F]F^-^ from supplier 1 was 48.4 ± 8.3% non-decay corrected (*n* = 14), while the mean synthesis yield obtained with [^18^F]F^-^ from supplier 2 was 47.8 ± 8.9% non-decay corrected (*n* = 30). No statistical difference has been found between the two suppliers.

### 2.3. Quality Controls

#### 2.3.1. Acceptance Criteria

Since Monograph 07/2021:3116 “PSMA-1007(^18^F) injection” [13] was not published in Eu. Pharm.at the time of IMPD drafting, the control of the investigational medicinal product should be properly described. Acceptance criteria, specifications and release timing were chosen in compliance with the general texts and monographs of the current European Pharmacopoeia and are summarized in Table 4.

The validation of analytical procedures, the acceptance limits and the parameters (specificity, linearity, range, accuracy, precision, quantification and detection limit) for performing validation of analytical methods has been carried out according to the ICH guideline Q2(R1) [14] and described in the IMPD, in the proper section.

#### 2.3.2. Batch Analysis and Process Validation

Process validation has been carried out by producing 3 different batches for each [^18^F]F^-^ supplier, with an [^18^F]F-PSMA-1007 activity in the range reported in the acceptance criteria.

Batch analysis, batch results for representative batches for each supplier are shown in Table 5.

#### 2.3.3. Stability

Another important section of IMPD is related to Stability. In Section 2.2.1, P.8 of the IMPD the shelf life and storage of the IMP should be defined based on the stability profile of the active substance and the available data on the IMP. We assessed stability tests at one, three, and six hours for all three validation batches as described in Table 6.

## 3. Discussion

Preparation of ^18^F-radiopharmaceuticals is usually accomplished in radiopharmacy with an on-site cyclotron. The external supply of [^18^F]F^-^ is usually hampered by several economic and logistic factors but one of the principal hurdles to overcome is to demonstrate to the Competent Authority that the quality of [^18^F]F^-^ from an external supplier is suitable for obtaining a radiopharmaceutical with defined quality requirements.

These results confirmed the same level of quality of [^18^F]F^-^ from both suppliers and this is very important considering that the [^18^F]F^-^ was produced using the same target material and foils but different proton beam energy (16.5 MeV for supplier 1 vs. 19 MeV for Supplier 2) and beam currents (140 µA for supplier 1 vs. 300 µA for Supplier 2).

The different delivery time of the radionuclide had no impact on radionuclidic impurity profile and on radiochemical purity.

[^18^F]F^-^ from both supplier had the same quality characteristics in term of radionuclidic purity and did not impact the radiochemical yield of the labelling reaction. The acceptance criteria for the quality control parameters of the IMP were fully met by fluoride from both manufacturers.

Bioburden and sterility and stability of the IMP [^18^F]F-PSMA-1007 was not affected by [^18^F]F^-^ from both suppliers.

## 4. Materials and Methods

### 4.1. Description of [^18^F]F-PSMA-1007 Manufacturing Process

#### 4.1.1. Set up of Radiosynthesizer

**Figure 3:**
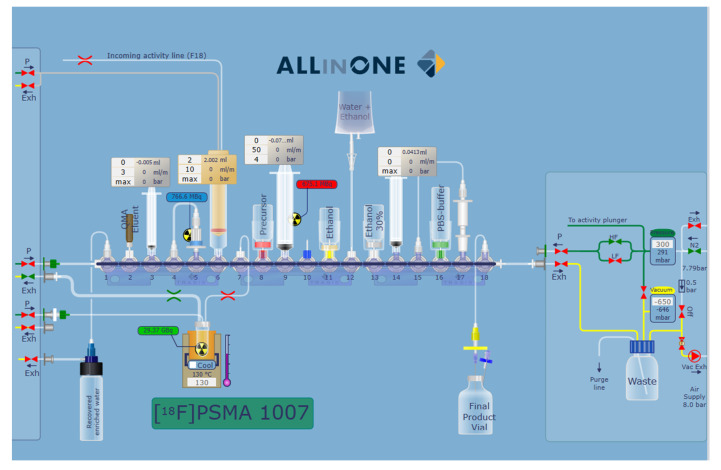
User graphic interface of the synthesis cassette for the preparation of [^18^F]F-PSMA-1007.

The radiosyntheses were conducted on an AllInOne PET tracer cassette radiosynthesizer (TRASIS SA-Rue Gilles Magnée 90 4430, Ans, Belgium). The module is placed in a shielded cell (Elena Beta, COMECER S.p.A, Castelbolognese, Italy) located in a cleanroom environment. User graphic interface of the synthesis process is shown in Figure 3.

No changes were made to the production process developed and established by ABX GmbH in collaboration with TRASIS SA (Rue Gilles Magnée 90 4430, Ans, Belgium). The only change was a modification of the synthesis module, consisting of the replacement of the fluoride inlet tube to allow the removal of the fluoride from the vial coming from the supplier. This change was evaluated with TRASIS and approved during the validation runs.

#### 4.1.2. Reagents

Reagents are described in Table 7. [^18^F]F^-^ was produced by AAA (Advanced Accelerator Applications a Novartis Company—via Piero Maroncelli 40, 47014 Meldola (FC), Italy (Supplier 1), and by Radiopharmacy & Cyclotron Dept. IRCCS Ospedale Sacro Cuore-Don Calabria (Negrar di Valpolicella), Italy (Supplier 2).

The production of [^18^F]F^-^ was carried out by a PET Trace cyclotron (supplier 1) and by an ACSI-TR19 cyclotron (supplier 2) by irradiation of the target material (H_2_^18^O) with a proton beam, according to the nuclear reaction ^18^O (p, n) ^18^F. Both cyclotrons were equipped with a Niobium (Nb) target with Havar foils. Single target has been irradiated by both suppliers; target volumes were 1.6 mL for supplier 1 and 2.8 mL for supplier 2. Proton beam has E_max_ = 16.5 MeV, with a beam current up to a 140 µA (supplier 1), E_max_= 19 MeV, with a beam current up to a 300 µA (supplier 2). Transport of target material was carried out by Helium pressure from the target to a borosilicate glass vial with crimped silicone closure.

Reagent Kit “Reagent for the synthesis of [^18^F]F-PSMA-1007 was produced and supplied by ABX GmbH—Advanced Biochemical Compounds (Radeberg, Germany). Kit contains the precursor PSMA-1007 (manufactured under GMP) and all reagents for the radiosynthesis contained in sterile vials. Single-use sterile cassettes were supplied by TRASIS SA.

#### 4.1.3. Process Description

The detailed information on every single step, not available from the module manufacturer, indicating the components used for each step including any relevant in-process controls should be provided to the Regulatory Agency. The production process is described below:

Transfer of the [^18^F]F^-^ in aqueous solution from the vial onto the Sep-PAK Light QMA cartridge anion exchange pre-purification cartridge. The Sep-PAK Light QMA cartridge retains the anion [^18^F]F^-^ but not cationic metallic impurities which are eliminated in the waste;

Recovery of the [^18^F]F^-^ from the Sep-PAK Light QMA cartridge by elution with TBAHCO_3_ solution (aqueous solution stabilized with ethanol). The anion HCO_3_^−^ elutes the anion [^18^F]F^-^ and the TBA^+^ acts as a phase transfer catalyst in the subsequent S_N_2 nucleophilic substitution reaction. The eluted [^18^F]F^-^ is directed into the reactor where it is evaporated to dryness;

The PSMA-1007 precursor dissolved in 2 mL of DMSO is added to the reactor; the choice of DMSO resides on its aprotic characteristics and high boiling point.

The reactor temperature is set at 105 °C and it is continuously monitored;

The radiolabelling reaction time is 600 s;

At the end of the radiolabelling reaction, the reactor content is recovered and purified through a cartridges system, consisting of a PS-H+ cartridge cation exchanger stacked on a C18ec cartridge. The PS-H+ cation exchanger has the function of retaining cationic impurities such as tetrabutylammonium. The C18ec cartridge is made up of derivatized silica with C18 chains. The C18ec cartridge is a substantially apolar type cartridge and has the function of purifying the solution from polar type impurities. It retains [^18^F]F-PSMA-1007 which will then be eluted with a 30% ethanol mixture. The polar impurities and DMSO are therefore not retained but directed towards the waste;

The cartridge system is then washed with 22 mL of an aqueous solution of 10% ethanol which is then directed into the waste and flushed with medical air;

The radiopharmaceutical is then eluted from the cartridge system with 8 mL of an aqueous solution of 30% ethanol, sterilized using a 0.22 µm sterilizing filter (Cathivex-GV filter, 0.22 µm, PVDF, sterile) and collected in the product vial;

The radiopharmaceutical is now buffered by adding phosphate buffer saline, dispensed through the 0.22 µm sterilizing filter (Cathivex-GV filter, 0.22 µm, PVDF, sterile).

At the end of the synthesis, the product vial activity is measured in a dose calibrator for the verification of the expected activity and the calculation of the activity concentration.

### 4.2. Quality Control

#### 4.2.1. Standard Procedures

The pH value of the formulation was determined by pH strips (Merck pH indicator strip, Acilit, increment 0.5 pH unit). TBA was measured by TLC according to the European Pharmacopoeia monograph 07/2021:20433 [15].

The Endotoxin test was performed by the Limulus amebocyte lysate test (LAL test) on an Endosafe Nexgen-PTS™ (Charles River Laboratories Italia, 26866 Sant’Angelo Lodigiano (LO), Italy)

As required by national regulations on quality assurance (NBP MN), since this is a preparation that cannot be subjected to terminal sterilization, it should be sterilized by filtration with a sterile disposable membrane having pores of nominal diameter 0.22 microns. In particular, sterile, Polyvinylidene difluoride (PVDF) Cathivex-GV (Merk Millipore, Darmstadt, Germany) filter filters, 0.22 µm, PVDF, compatible with ethanolic solution, are used for sterilization.

Determination of suitability and the bioburden of the pre-filtration product were carried out (Eurofins Laboratory Biolab Srl, Vimodrone, Milan), using 1 mL for each test sample;

The results were the following:

TAMC (total aerobic microbial count) < 1 cfu/mL and

TYMC (Total Yeast and Mold count) <1 cfu/mL, where < 1 cfu/mL means absence of colonies.

According to NBP MN, the filter integrity has been checked by bubble point test before the release of the lot (Integritest 4 system, Merck Millipore, Merck KGaA ©, Darmstadt, Germany) was used for this verification.

Sterility was tested post-release by an independent institution, using 1 mL for each test sample with direct inoculation of the culture medium, according to current European Pharmacopoeia Monograph 2.6.1 Sterility.

#### 4.2.2. HPLC Analysis

Standard ^nat^F-PSMA-1007 and OH-PSMA-1007 and PSMA-1007 were purchased from ABX GmbH—Advanced Biochemical Compounds (Radeberg, Germany)

HPLC analysis was performed on an Ultimate 3000 system equipped by a UV variable wavelength detector RS300 (Thermo Fischer Scientific, Germany) and a radiometric detector (GABI, Raytest, Germany). The system was controlled by Chromeleon software version 7.2 SR5 (Dionex Sunnyvale, CA, USA).

The column was an XTERRA RP18 5 µm 4.6 × 250 mm (Waters CorporationMilford, MA, USA). For the analysis a multi-step gradient was applied using solvent A (Acetonitrile) and solvent B (0.1% TFA in water): 20% A to 30% A in 2 min, then stable for a further 15 min, then from 30% A to 95% A for a further 6 min, and back to 20% A in 2 min, then stable for 10 min. The flow rate was set at 1 mL/min, UV wavelength at 225 nm. Column Oven: OFF. Injection volume 20 µL.

#### 4.2.3. Thin-Layer Chromatography (TLC)

TLC was performed using TLC Silica Gel 60 (Merck KGaA, Darmstadt, Germany), Approximately 1–2 microliters of IMP [^18^F]PSMA-1007 injection solution was spotted on the plate. The solvent for the development of the TLC plates was Acetonitrile/Water 3:2 *v*/*v*. The developed plate was analyzed by autoradiography on MS (MultiSensitive) storage phosphor screens and by a Cyclone Plus Storage Phosphor System (PerkinElmer).

#### 4.2.4. Gas-Liquid Chromatography (GLC)

Determination of DMSO and ethanol was carried out by GLC, on a TRACE GC^TM^ 1310 (Thermo Fischer Scientific, Germany) gas chromatograph equipped with an automatic headspace injector. The column was a DB624 (1.8 µm, 0.32 mm, 30 m), carrier gas was nitrogen and the detector was a flame ionization detector (FID) set at 280 °C. The oven temperature was programmed from 40 °C to100 °C in 6 min (Ethanol), 40 °C to 150 °C in 16 min (DMSO). System was controlled by Chromeleon software.

## 5. Conclusions

This study confirms the possibility of preparing F-18 radiopharmaceuticals in centers without cyclotron. The results clearly demonstrate that many existing preconceptions could be overcome by applying a well-implemented quality assurance system that clearly defines acceptance criteria, validations plans and methods for quality control. The problem of radionuclidic impurities is often overestimated by the regulatory authority: our results demonstrate the feasibility of using external suppliers of [^18^F]F^-^ for preparing experimental radiopharmaceuticals, provided that a well-established quality assurance system has been correctly implemented. These results can be encouraging for those radiopharmacies that intend to expand their preparations towards new radiopharmaceuticals even if they do not have a cyclotron and do not foresee such an important investment.

## Figures and Tables

**Table 1:** Possible impurities deriving from irradiation with protons of Havar foil.

Radionuclide	T1/2	Reaction	Decays to
^55^Co	17.5 h	^58^Ni (p, α) ^55^Co	^56^Fe, stable
^56^Co	78.8 days	^56^Fe (p, n) ^56^Co	^56^Fe, stable
^57^Co	271.7 days	^60^Ni (p, α) ^57^Co ^57^Fe (p, n) ^57^Co	^56^Fe, stable
^58^Co	70.8 days	^58^Fe (p, n) ^58^Co	^58^Fe, stable
^51^Cr	27.7 days	^52^Cr (p, pn) ^51^Cr	^57^Cr, stable
^54^Mn	312.2 days	^54^Cr (p, n) ^54^Mn	^54^Cr, stable
^52^Mn	5.6 days	^52^Cr (p, n) ^52^Mn	^52^Cr, stable
^48^V	16.0 h	^48^Ti (p, n) ^48^V	^48^Ti, stable
^95m^Tc	61 days	^95^Mo (p, n) ^95m^Tc	^95^Mo, stable

**Table 2:** Quality control data on [^18^F]F^-^ batches from different suppliers.

Parameter	Method	Acceptance Criteria	Batch 14/02/19 Supp. 1	Batch 15/03/19 Supp. 1	Batch 12/04/19 Supp. 1	Batch 09/07/20 Supp. 2	Batch 30/07/20 Supp. 2	Batch 06/08/20 Supp. 2
[^18^F]F^-^ Activity	Dose calibrator	12,690–30,600 MBq	12,691 MBq	19,721 MBq	30,599 MBq	16,562MBq	20,139 MBq	21,281 MBq
Radioactive Concentration	Dose calibrator	2.7–21.8 GBq/mL	9.1 GBq/mL	13.1 GBq/mL	21.8 GBq/mL	2.76 GBq/mL	3.36 GBq/mL	3.55 GBq/mL
Volume		1.4–6	1.4 mL	1.5 mL	1.4 mL	6 mL	6 mL	6 mL
Appearance	Visual test	Clear and colorless solution	Complies	Complies	Complies	Complies	Complies	Complies
Identification	γ Spectrometry	Peaks at 511 and 1022 Kev	Complies	Complies	Complies	Complies	Complies	Complies
	Half life	105–115 min	Complies	Complies	Complies	Complies	Complies	Complies
Identification	HPLC	T_R_ [^18^F]F- test ± 0.5 min T_R_ [^18^F]F^-^ reference standard	Complies	Complies	Complies	Complies	Complies	Complies
Radiochemical Purity	HPLC	[^18^F]F^-^ ≥ 98.5%	99.6%	99.2%	99.5%	100%	100%	100%
pH	pH strips	8–14	9	9	9	9	9	9
Radionuclidic Purity	γ Spectrometry	Impurities < 0.1%	Complies	Complies	Complies	Complies	Complies	Complies
Bacterial Endotoxins	Eur.Ph.	≤20 EU/V	≤10 EU/V	≤10 EU/V	≤10 EU/V	≤10 EU/V	≤10 EU/V	≤10 EU/V

**Table 3:** Batch formula of [^18^F]F-PSMA-1007.

Components	Function	Amount/Activity
[^18^F]F-PSMA-1007	Active Pharmaceutical Ingredient (API)	7000–18,200 MBq Activity Reference Time (ART)
Phosphate Buffer Saline (ready for use): Na_2_HPO_4_ KH_2_PO_4_ NaCl KCl	Buffer solution	17.229 mg 3.0 mg 120.0 mg 3.0 mg 15 mL Water for Injection
Ethanol 30%	Eluent	8 mL

**Table 4:** Recommended test for the quality controls and pre/post-release time schedule.

Parameter	Method	Acceptance Criteria	Pre/Post Release
[^18^F]F-PSMA-1007 Activity	Dose calibrator	7000–18,200 MBq	pre
Radioactive concentration	Dose calibrator	300–791 MBq/mL	pre
Volume		23 mL	pre
Appearance	Visual test	Clear and colorless solution	pre
Identification	γ-spectrometry	Peaks at 0.511 Mev and 1.022 Mev	pre
	Half-life	105–115 min	pre
Identification	HPLC	T_R_ [^18^F]F-PSMA-1007 ± 0.5 min T_R_ PSMA-1007 reference standard	pre
Radiochemical Purity	TLC	[^18^F]F^-^ ≤ 5% [^18^F]F-PSMA-1007 ≥ 95%	pre
Radiochemical Purity	HPLC	[^18^F]F^-^ ≤ 5% [^18^F]F-PSMA-1007 ≥ 95%	pre
Chemical Purity	HPLC	PSMA-1007 ≤ 0.1 mg/V_max_ Sum of impurities ≤ 0.5 mg/V_max_	pre
	TLC	TBA ≤ 260 μg/mL	pre
Ethanol	GLC	≤10% P/V	pre
DMSO	GLC	≤5000 ppm	pre
pH	pH strips	4.8–8.5	pre
Filter Integrity	Bubble Point Test	≥50 psi	pre
Radionuclidic Purity (Impurities > 2 h Half Life)	γ-Spectrometry	≤0.1%	post
Sterility	Sterility Test (Eur.Ph.)	Sterile	post
Bacterial Endotoxins	Eur.Ph.	≤175 EU/V	pre

**Table 5:** Results of [^18^F]F-PSMA-1007 representative batches obtained with [^18^F]F^-^ from different suppliers.

Parameter	Method	Acceptance Criteria	Batch 14/02/19 Sup. 1	Batch 15/03/19 Sup. 1	Batch 12/04/19 Sup. 1	Batch 09/07/20 Sup. 2	Batch 30/07/20 Sup. 2	Batch 06/08/20 Sup. 2
[^18^F]F-PSMA-1007 Activity	Dose Calibrator	7000–18,200 MBq	7030 MBq	9990 MBq	18,200 MBq	7600 MBq	8800 MBq	10,260 MBq
Radioactive Concentration	Dose Calibrator	300–791 MBq/mL	305.6 MBq/mL	434.3 MBq/mL	791 MBq/mL	330 MBq/mL	382 MBq/mL	446 MBq/mL
Volume		23 mL	23 mL	23 mL	23 mL	23 mL	23 mL	23 mL
Appearance	Visual test	Clear and Colorless Solution	Complies	Complies	Complies	Complies	Complies	Complies
Identification	γ-Spectrometry	Peaks at 0.511 Mev and 1.022 Mev	Complies	Complies	Complies	Complies	Complies	Complies
	Half-Life	105–115 min	Complies	Complies	Complies	Complies	Complies	Complies
Identification	HPLC	T_R_ [^18^F]F-PSMA-1007 ± 0.5 min T_R_ PSMA-1007 Reference Standard	13.5 min	13.5 min	13.6 min	13.5 min	13.5 min	13.6 min
Radiochemical Purity	TLC	[^18^F]F^-^ ≤ 5% [^18^F]F-PSMA-1007 ≥ 95%	0.3% 99.7%	0.9% 99.1%	0.2% 99.8%	0.7% 99.3%	0 100%	0.6% 99.4%
Radiochemical Purity	HPLC	[^18^F]F^-^ ≤ 5% [^18^F]F-PSMA-1007 ≥ 95%	n.p. 98.3%	n.p. 98.1%	n.p. 97.7%	n.p. 97.9%	n.p. 98.5%	n.p. 98%
Chemical Purity	HPLC	PSMA-1007 ≤ 0.1 mg/V_max_ Sum of Impurities ≤ 0.5 mg/V_max_	Complies	Complies	Complies	Complies	Complies	Complies
	TLC	TBA ≤ 260 μg/mL	Complies	Complies	Complies	Complies	Complies	Complies
Ethanol	GLC	≤10% P/V	Complies	Complies	Complies	Complies	Complies	Complies
DMSO	GLC	≤5000 ppm	Complies	Complies	Complies	Complies	Complies	Complies
pH	pH Strips	4.8–8.5	7.5	7.5	7.5	7.5	7.5	7.5
Filter Integrity	Bubble Point Test	≥50 psi	≥50 psi	≥50 psi	≥50 psi	≥50 psi	≥50 psi	≥50 psi
Radionuclidic Purity	γ-spectrometry	≤0.1%	Complies	Complies	Complies	Complies	Complies	Complies
Sterility	Sterility Test (Eur.Ph.)	Sterile	Sterile	Sterile	Sterile	Sterile	Sterile	Sterile
Bacterial Endotoxins	Eur.Ph.	≤175 EU/V	≤10 EU/V	≤10 EU/V	≤10 EU/V	≤10 EU/V	≤10 EU/V	≤10 EU/V

**Table 6:** Stability test of injectable solution of [^18^F]F-PSMA-1007.

**1 h Stability Test**								
**Parameter**	**Method**	**Acceptance Criteria**	**Batch 14/02/19** **Sup. 1**	**Batch 15/03/19** **Sup. 1**	**Batch 12/04/19** **Sup. 1**	**Batch 09/07/20** **Sup. 2**	**Batch 30/07/20** **Sup. 2**	**Batch 06/08/20** **Sup. 2**
Appearance	Visual test	Clean and Colorless Solution	Complies	Complies	Complies	Complies	Complies	Complies
Radiochemical Purity	TLC	[^18^F]F^-^ ≤ 5% [^18^F]F-PSMA-1007 ≥ 95%	0.5% 99.5%	1.2% 98.8%	0.5% 99.5%	0.7% 99.3°%	0.2% 98.8°%	0.6% 99.4%
Radiochemical Purity	HPLC	[^18^F]F^-^ ≤ 5% [^18^F]F-PSMA-1007 ≥ 95%	n.p. 98.4%	n.p. 97.8%	n.p. 97.7%	n.p. 97.9%	n.p. 98.3%	n.p. 98.2%
Chemical Purity	TLC	TBA ≤ 260 µg/mL	Complies	Complies	Complies	Complies	Complies	Complies
pH	pH Strips	4.8–8.5	7.5	7.5	7.5	7.5	7.5	7.5
**3 h stability test**								
**Parameter**	**Method**	**Acceptance Criteria**	**Batch 14/02/19** **Sup. 1**	**Batch 15/03/19** **Sup. 1**	**Batch 12/04/19** **Sup. 1**	**Batch 09/07/20** **Sup. 2**	**Batch 30/07/20** **Sup. 2**	**Batch 06/08/20** **Sup. 2**
Appearance	Visual Test	Clean and Colorless Solution	Complies	Complies	Complies	Complies	Complies	Complies
Radiochemical Purity	TLC	[^18^F]F^-^ ≤ 5% [^18^F]F-PSMA-1007 ≥ 95%	0.4% 99.6%	1.3% 98.7%	0.6% 99.4%	0.8% 99.2%	0.2% 99.8%	0.7% 99.3%
Radiochemical Purity	HPLC	[^18^F]F^-^ ≤ 5% [^18^F]F-PSMA-1007 ≥ 95%	n.p. 98.4%	n.p. 97.8%	n.p. 97.7%	n.p. 97.9%	n.p. 98.1%	n.p. 98.1%
Chemical Purity	TLC	TBA ≤ 260 µg/mL	Complies	Complies	Complies	Complies	Complies	Complies
pH	pH Strips	4.8–8.5	7.5	7.5	7.5	7.5	7.5	7.5
**6 h stability test**								
**Parameter**	**Method**	**Acceptance Criteria**	**Batch 14/02/19** **Sup. 1**	**Batch 15/03/19** **Sup. 1**	**Batch 12/04/19** **Sup. 1**	**Batch 09/07/20** **Sup. 2**	**Batch 30/07/20** **Sup. 2**	**Batch 06/08/20** **Sup. 2**
Appearance	Visual Test	Clean and Colorless Solution	Complies	Complies	Complies	Complies	Complies	Complies
Radiochemical Purity	TLC	[^18^F]F^-^ ≤ 5% [^18^F]F-PSMA-1007 ≥ 95%	0.4% 99.6%	1.9% 98.1%	0.9% 99.1%	09% 99.1%	0.3% 99.7%	0.8% 99.2%
Radiochemical Purity	HPLC	[^18^F]F^-^ ≤ 5% [^18^F]F-PSMA-1007 ≥ 95%	n.p.98.4%	n.p. 97.8%	n.p. 97.7%	n.p. 97.7%	n.p. 98 %	n.p. 98.1%
Chemical Purity	TLC	TBA ≤ 260 µg/mL	Complies	Complies	Complies	Complies	Complies	Complies
pH	pH Strips	4.5–8.5	7.5	7.5	7.5	7.5	7.5	7.5

**Table 7:** Description of the synthesis reagents.

	Components	Amount	Supplier
A	[^18^F]F^-^	12,690–30,600 MBq	Supplier 1 Supplier 2
B	Kit reagent “Reagent for synthesis of [^18^F]F-PSMA-1007”:		
	PSMA-1007 precursor	1.6 mg	ABX Advanced biochemical compounds
	DMSO for precursor	2.2 ± 0.1 mL	
	Ethanol	8 ± 0.3 mL	
	Phosphate buffered Saline	15 ± 0.5 mL	
	30% Ethanol solution	8.5 ± 0.3 mL	
	Ethanol for Water bag	5.7 ± 0.2 mL	
	Water for injections (B.Braun)	100 mL ± 10%	
	Tetrabutylammonium Hydrogen Carbonate (0.075 M)	750 ± 20 µL	
	C18ec cartridge Chromabond^®^ (Macherey Nagel)	1	
	PS-H+ cartridge	1	
	Sep-PAK Light QMA cartridge	1	
	Syringe, 3 mL, empty, sterile (Becton Dickinson BD)	1	
	Syringe, 10 mL, empty, sterile (Becton Dickinson BD)	1	
	Cathivex-GV filter, 0.20 µm, PVDF, sterile (EMD Millipore)	1	
	MILLEX-25 filter 0.20 µm, sterile (EMD Millipore)	1	
C	Cassette ^18^F-PSMA 1007	1	TRASIS SA

## Data Availability

The data presented in this study are available on request from the corresponding author.
